# Molecular and Genomic Profiling of Lung Cancer in the Era of Precision Medicine: A Position Paper from the Italian Association of Thoracic Oncology (AIOT)

**DOI:** 10.3390/cancers12061627

**Published:** 2020-06-19

**Authors:** Nicola Normanno, Massimo Barberis, Filippo De Marinis, Cesare Gridelli, on the behalf of the AIOT Expert Panel

**Affiliations:** 1 Cell Biology and Biotherapy Unit, Istituto Nazionale Tumori, “Fondazione G. Pascale”—IRCCS, 80131 Napoli, Italy; 2 Department of Pathology, European Institute of Oncology, 20141 Milan, Italy; massimo.barberis@ieo.it; 3 Division of Thoracic Oncology, European Institute of Oncology, IRCCS, 20141 Milan, Italy; filippo.demarinis@ieo.it; 4 Division of Medical Oncology, “S. G. Moscati” Hospital, 83100 Avellino, Italy; cgridelli@libero.it

**Keywords:** precision medicine, comprehensive genomic profiling, non-small-cell lung cancer, biomarkers, clinical practice

## Abstract

The identification of the optimal cancer treatment has become progressively more intricate for non-small-cell lung cancer (NSCLC) patients due to the multitude of options available. The testing of biomarkers to predict clinical responses to therapies is pivotal to stratify the patients based on the molecular features of their tumors. The number of actionable genetic alterations to be tested is increasing together with the comprehension of the molecular mechanisms underlying tumor growth and development. The possibility of using next generation sequencing-based approaches enhanced the acquisition of genetic data with potential clinical usefulness, and favored the integration of precision medicine in clinical practice. The availability of targeted sequencing panels that cover genetic alterations in hundreds of genes allows the performance of a comprehensive genomic profiling (CGP) of lung tumors. However, different issues still need to be solved, from the tissue needed for next generation sequencing analysis, to the choice of the test and its interpretation in the clinical context. This position paper from the Italian Association of Thoracic Oncology (AIOT) summarizes the results of a discussion from a Precision Medicine Panel meeting on the challenges to bringing CGP and, therefore, precision medicine into the daily clinical practice.

## 1. Introduction

The selection of the most appropriate cancer treatment for lung cancer patients has become more complex due to the variety of options available. The possibility to design drugs targeting the specific vulnerabilities of cancer cells and tailor the treatment on the basis of the molecular characteristics of the tumor, defined by biomarkers, constitutes the basis of precision medicine. Biomarker testing is able to identify subgroups of patients that are more likely to benefit from a therapy. This indication is important for the clinicians to unravel the complexity of treatment decisions. Now, a number of biomarkers in non-small-cell lung cancer (NSCLC) are being tested in routine diagnostics, including EGFR and BRAF mutations, as well as ALK and ROS1 rearrangements and PD-L1 expression [1]. The use of biomarkers to identify population subgroups with impressive responses to targeted therapies, as observed in ALK-rearranged and ROS1-rearranged NSCLC, for which crizotinib was associated with remarkably prolonged survival [2], and in NSCLC carrying EGFR activating mutations, which are known to increase sensitivity to EGFR tyrosine-kinase inhibitors (TKI) [3,4]. The list of actionable alterations and thus the number of biomarkers to be tested is going to increase proportionally in the near future, together with the comprehension of the molecular mechanisms of tumor growth [5]. In lung adenocarcinoma, oncogenic drivers that offer the possibility of therapeutic intervention have been identified in over 50% of the cases [6,7]; however, the fraction of lung cancer patients who may benefit from targeted treatments might further increase.

The development of precision medicine owes part of its progress to the huge technological advances in sequencing we have seen in the last decades. The introduction of Next Generation Sequencing (NGS) reinvented translational research and determined an important expansion of genomic studies. Currently, the presence on the market of a plethora of NGS approaches for genomic profiling has enhanced the acquisition of genetic data with potential clinical usefulness [8]. Moreover, this application is not limited to the analysis of tumor tissue, but is now open also to the use of liquid biopsies, i.e. plasma, urine and cerebrospinal fluids. The availability of targeted sequencing panels that cover genetic alterations in hundreds of genes allows the performance of a comprehensive genomic profiling (CGP) of the tumor of each individual patient in a single analysis.

CGP can provide relevant information on actionable mutations, including rare variants, but also data that might also be important for immunotherapy. Although immune checkpoint inhibitors (ICIs) have represented a major breakthrough in lung cancer therapy, a majority of patients do not benefit from this approach and, therefore, biomarkers are definitely needed for better patient stratification. In this regard, CGP can provide information on tumor mutation burden (TMB) and on several genetic alterations that might affect the efficacy of ICIs [9].

The above-summarized evidence suggests that the implementation of precision medicine in the clinical practice and in the clinical research of lung cancer does require CGP of the tumor. However, in order to bring CGP and therefore precision medicine into our daily clinical practice, it is necessary to define a clear path that goes from the tissue needed for next generation sequencing analysis, to the choice of the test and its interpretation in the clinical context. This position paper from the Italian Association of Thoracic Oncology (AIOT, Associazione Italiana di Oncologia Toracica) summarizes the results of a discussion from a Precision Medicine Panel meeting on the challenges to implementing precision medicine in the path of treatment of patients with lung cancer (Appendix A). The meeting gathered different professionals: oncologists, pathologists, molecular biologists, interventional radiologists and pulmonologists. The discussed topics were: (1) the sample, (2) the test and (3) clinical applicability.

## 2. The Samples

The diagnostic approach to pulmonary carcinomas in the era of targeted therapies and immunotherapy requires both the definition of the histological type and the analysis of specific molecular biomarkers. 

In many hospitals within Italy, Surgical Pathology services and/or Molecular Genetics are already able to offer a molecular makeup of the biopsies and cytological samples. Indeed, the number of drugs that will be available in clinical practice is increasing—and, for those compounds already in the clinics, combinations or sequences of drugs are being approved—in order to overcome the development of resistance mechanisms, which are common events with targeted therapies. 

The current challenge for the pathologists is indeed represented by the necessity to go beyond the histology-based diagnosis, to provide data to guide treatment choice in a time-effective and precise manner, trying also to comply with the criterion of economic sustainability. Therefore, this constitutes a demanding task that collides with many difficulties. One of the main problems is that, in most patients, lung cancer is diagnosed at advanced stages from small biopsies or cytological samples. Such samples can represent an additional difficulty both for the cytological-histological diagnostic assessment and for the molecular evaluation. In particular, the inadequacy of the material due to the absence or inadequate quantity of tumor cells, the depletion of the material due to repeated immunocytochemical assessments for the diagnosis of the histological type and the interference with stromal and/or inflammatory cells represent major challenges [10]. Different critical points emerged from the collegial discussion, mainly related to the advisory role of the Pathologists of the panel. The absence of pathologists with specific expertise in the field of pulmonary oncology is widely known. In those situations where this problem is less marked, a lack of operational procedures for the management of small biopsies might be registered. Dedicated protocols, widely available in the scientific literature, are needed for the correct handling of small samples to enable a thrifty use of the material for biomarker testing and the right management of molecular analyzes [11].

Another aspect that emerged from the discussion is the extreme diversity of organizational systems, of the available technological platforms and of the levels of training of the staff dedicated to these activities. Difficulties were also reported in organizing multidisciplinary working groups with pulmonologists, oncologists, interventional radiologists and surgeons. The current complexity of this activity, although increasing the opportunities for the patients, generates zones of inefficiency, loss of precious time for assistance and waste of resources.

The discussion could also benefit from the pivotal contributions from interventional radiologists and pulmonologists. In particular, the crucial matters and chances offered by sampling techniques by means of transcutaneous needle biopsies guided by ‘imaging’ were discussed. The core-needle biopsy, when used by expert hands, provides greater sensitivity, specificity and accuracy compared to fine needle aspiration, with no increased risk of complications [12]. Parameters identified as important were patient orientation and compliance, the access and the type of needle chosen, the expertise of the sampler and the ability to manage possible complications (for example pneumothorax, bleeding, air embolism).

An extensive discussion was also carried out on endobronchial ultrasound-guided transbronchial needle aspiration (EBUS-TBNA), a technique that has gained extensive approval in the diagnosis and mediastinal staging of lung tumors [13]. This technique offers excellent levels of diagnostic accuracy, while on the other hand some uncertainty still exists regarding its performance for tumor genotyping. In fact, the various studies published are difficult to reproduce for different reasons: some studies included the determination of the mutational status of single genes, others assessed more or less extensive panels of genes to evaluate hotspot mutations, and others chose panels to also evaluate fusion genes and copy-number variations [14,15,16]. Other dissimilarities consisted in a difference in the number of needle passes per single target, as well as in the number of lymph nodes evaluated; the size of the needle used; the sensitivity of the assay chosen for molecular evaluation; and finally in the decision to adopt rapid onsite evaluation (ROSE). In theory, ROSE should help overcome the possibility of obtaining inadequate samples [17]. It is used in centers with high volumes of activity to maximize the percentage of patients diagnosed and genotyped in the same bronchoscopic session. It has been shown that the benefit of ROSE is related to the increase in the number of adequate samples for the diagnostic definition, but not for the molecular evaluation. Indeed, if the ROSE approach documents that the sample consists predominantly of necrosis, lymphocytes and a minimal amount of tumor cells, the sampler can change its sampling strategy to achieve better results. Currently, the EBUS-TBNA approach provides a cytological sample, because the practice of core biopsy is not so common, despite being feasible. This limit should be overcome, particularly because the absence of a histological sample might limit patients’ opportunity to access clinical trials, where cellular blocks are not accepted.

Over the last few months, as has happened for other expensive technologies, there has been a rapid escalation in the acquisition of the technologies required to start up the EBUS-TBNA activity. Unfortunately, it did not result in an increased efficiency of the system, because little importance was dedicated to the training of the staff, who can reach adequate levels of autonomy by actively attending centers with high productivity.

In conclusion, the discussion carried out between experts on the best management of the sample identifies the following objectives that should be improved/achievable (Table 1):

- Centralization of the techniques which are more complex to implement;
- Optimization of the resources, always keeping an eye on the economy of scale;
- Need to implement multidisciplinary activities;
- Improvement of the hands-on training activities;
- Activation of the procedures needed to make the figure of the pathologist with specific expertise in the field of lung cancer more widespread.

## 3. The Test

A second panel of experts focused on the status of the art, the difficulties and the implementations needed for the molecular profiling of lung cancer samples, with a specific focus on the implementation of large NGS panels for CGP. The topics that were discussed are the following (Table 1):

- Informed consent and privacy.
- Tissue versus liquid biopsy.
- CGP technologies: sequencing of the whole exome versus targeted sequencing panels.
- Organization of a laboratory that performs CGP tests.
- Outsourcing or in-house testing.
- Validation and verification of CGP tests.
- Interpretation of CGP test results (integration of data in the clinical context).
- Management of incidental findings.
- Mutational report: structure and minimum information to be included.

### 3.1. Informed Consent and Privacy

The first requirement before any approach to a molecular test is informed consent, which should be understood and signed by the patient. The panel of experts anticipates that a shared format of consensus will be created at the regional/national level, in order to allow harmonization. Consent should be clear and comprehensive, in order to cover and explain to the patient every step that will be taken, and must include permission for the management, analysis and storage of the material for a comprehensive characterization of the tumor (either morphological, phenotypical or molecular). 

### 3.2. The Sample: Tissue vs Liquid Biopsy 

As previously specified, various types of tissue specimens can be used as starting material for mutation testing [18]. The use of tumor tissue for the identification of predictive biomarkers of response to targeted therapies is mandatory at diagnosis and onset, when this type of material should be considered as the golden standard and should not be replaced, at least in these phases, by liquid biopsy. In those situations where a real-time monitoring of therapies of the patient is required, liquid biopsy represents a valid alternative to tumor tissue. In fact, liquid biopsy better recapitulates the tumor heterogeneity, and could be useful for the evaluation of possible resistance mechanisms [19]. However, the use of liquid biopsy should be carefully evaluated, and more studies are needed in order to assess its accuracy. Indeed, challenges related to the limits of detection of the tests still exist, especially due to the possibility of false negative and false positive results [20]. In the event of a non-shedding tumor, the levels of circulating DNA that could be isolated from liquid biopsy can be below the limit of detection of the methods used for testing, thus lowering the chance to detect molecular alterations [19]. On the other hand, sequencing artifacts and clonal hematopoiesis of indeterminate potential (CHIP) might lead to false positive signals [21]. 

Currently, the evaluation of complex genetic alterations, such as fusions, copy number variations (CNV) or tumor mutation burden (TMB), by means of liquid biopsy requires accurate laboratory and clinical validation.

At present, liquid biopsy is mainly represented by plasma samples, but the use of other samples, such as cerebrospinal fluid, urine and pleural effusions, is being implemented as well.

### 3.3. Comprehensive Genomic Profiling Technologies

CGP is based on next generation sequencing approaches, and sequences millions of DNA fragments, allowing the analysis of hundreds of genes in parallel. Differences exist between the various available NGS approaches. For instance, whole exome sequencing (WES) allows the sequencing of the entire coding region of the genome; however, this technology is almost unfeasible in the clinics, where targeted sequencing panels covering a smaller number of genes are more prone to being implemented [22]. CGP should be considered only after appropriate clinical evaluation of patients and subsequent assessment of the adequacy of the sample, and should be performed in specialized laboratories. CGP panels should include the detection of single nucleotide variants (SNV), CNV and fusions. 

It is difficult to define the number of genes to be tested. Based on the drugs currently approved by the European Medicine Agency (EMA), the list is quite short: EGFR, BRAF, ALK, ROS1 and NTRK1. Many drugs targeting genetic alterations are in the advanced phases of clinical development, including agents targeting RET, MET and KRAS. However, the concept of CGP is wider, and it goes beyond what is already known, with the aim to improve the knowledge of the pathogenesis of cancer and personalize treatments. In this respect, for the assessment of the TMB, panels covering at least 1Mb of the coding region are required [23,24]. Therefore, we expect that CGP panels ideally cover over 300 genes. With the reduction of the sequencing costs, the use of these panels in routine clinical diagnostics should become more feasible.

Evidence suggests that targeted RNA sequencing can improve the detection of fusion genes compared with DNA sequencing [25]. A number of targeted sequencing panels using DNA and/or RNA to cover genetic alterations are commercially available from different vendors. 

### 3.4. Organization of a Laboratory Performing CGP Tests

Some prerequisites should be fulfilled in each laboratory performing or wanting to implement CGP testing. The structural adequacy of the laboratory must be verified: it is recommended to have dedicated areas with different spaces for each of the required steps of the analysis workflow, from extraction, analysis set-up and sequencing up to data storage [26].

In addition, the adequacy of the laboratory’s instrumental equipment should ensure the availability of latest-generation CE-IVD certification instruments and orthogonal validation technologies to confirm the obtained results. The adequacy of human resources is also desirable: it is important to ensure the presence of qualified and trained personnel (molecular biologists and bioinformatics).

Finally, standard operating procedures (SOPs) should be followed for the management and traceability of both samples and collected data.

### 3.5. Outsourcing or In-House Testing and CGP Validation/Verification

CGP could be performed by means of either in-house or outsource tests. Non-certified laboratory-developed tests must undergo an extensive validation, analyzing an adequate number of samples [26]. In the case of commercially-available, validated and CE-IVD tests, the quality of the procedures must be verified by performing internal quality checks, taking part in external quality control schemes and also with periodic verifications of the test performance.

Because of the costs of the equipment and the need of specialized personnel, an accurate evaluation should be performed by each center on the opportunity to outsource tests. If this will be the choice, these tests must ensure specific quality criteria to the requesting laboratories, and should thus provide:

- Public data on the failure rate.
- Public data on the validation procedures.
- Any other certification.
- Adherence to national and international external quality control schemes.

### 3.6. Interpretation of CGP Test Results

The conduction of a CGP test requires the introduction of the obtained results in the most appropriate clinical context. In this regard, it is necessary to implement molecular tumor boards (MTBs) for the collegial discussion of the results, especially for complex molecular data, in order to allow the identification of the best therapeutic approach in selected patients [27]. The MTB is a multidisciplinary team including treating physicians, and at least a molecular oncologist, a clinical geneticist and a molecular pathologist. The MTB report is issued after a collegial clinical discussion of the molecular results deriving from an in-house or outsourced test. The application of artificial intelligence systems is an important decision support tool for the evaluation of the CGP results. The interpretation of TMB results should take into account a number of variables, including the differences among available CGP panels. It might be better to classify the TMB results as high, intermediate and low, rather than using a fixed numerical cut-off that might be difficult to reproduce due to the inter-experimental variability of any assay.

### 3.7. Management of Incidental Findings

Large targeted sequencing panels for CGP often include genes that are possibly associated with hereditary cancer syndromes such as BRCA1/2, ATM and BAP1 etc. Different studies have reported that NGS for tumor molecular profiling can reveal likely secondary germline pathogenic and pathogenic variants [28]. Therefore, a dedicated pathway must be planned for patients with such incidental findings [29]. When testing tumor tissue, it is not possible to distinguish whether a mutation is somatic rather than germline without testing the matched normal tissue. Therefore, if a mutation potentially associated with hereditary cancer is detected during CGP, the patient should be referred to genetic counseling and possibly receive germline testing. 

### 3.8. Mutational Report: Structure and Minimal Required Information

A structured and comprehensive CGP report should include the following parts:

- A section for the pathological evaluation of the sample’s adequacy for testing.
- The list of genes included in the panel used for testing, detailing the coverage reached in the genes.
- The depth coverage reached by the sequencing run and the sensitivity limits of the panel.
- The allelic frequency of the variant(s) identified in the sample.
- The correct nomenclature of the alterations detected, which should follow the Human Genome Variation Society (HGVS) guidelines.

The report should also include an appropriate biological and pathological interpretation of the identified variants.

## 4. Clinical Applicability

The identification of genomic variants able to predict the response to targeted agents or to immunotherapy could reduce the use of unnecessary therapies, optimizing the treatment strategy. Nearly 90% of samples tested within the TGCA project harbor alterations in genes potentially associated with a predictive, prognostic, or diagnostic role [30,31]. The use of a molecular profile to guide therapeutic decisions could potentially improve clinical outcomes in approximately one-third of patients with advanced tumors [7]. Indeed, the NCCN guidelines do “strongly advises broader molecular profiling with the goal of identifying rare driver mutations for which effective drugs may already be available or to appropriately counsel patients regarding the availability of clinical trials” [32]. Focusing on lung adenocarcinoma, 20–25% of cancers carry actionable alterations for which targeted therapies are commercially available, but more than 40% of cancers display possible actionable alterations, as also suggested in CGP studies [33]. It is also known that lung adenocarcinoma is one of the tumors with a higher incidence of protein-altering mutations [34]. The scenario described above provides a rationale for the use of CGP upfront in patients with lung adenocarcinoma, which could detect actionable genetic alterations for which targeted drugs already exist in clinical practice and, even more likely, in clinical trials. The upfront use of targeted sequencing panels will also ensure that all the recommended biomarkers are tested in all patients. Although testing rates have significantly improved over time for most common biomarkers, such as EGFR and ALK, they are still suboptimal for novel targets, including ROS1 [35]. This phenomenon is due to several factors, including the fact that, in many cases, the tissue is not sufficient to cover the increasing number of approved biomarkers when using routine diagnostic techniques and sequential testing. 

Cost has been claimed as a possible limit for the implementation of upfront CGP. However, evidence suggests that the use of upfront NGS testing in patients with metastatic NSCLC is associated with significant cost savings and shorter time-to-test results [36].

CGP analysis can also identify mutations of unknown significance or driver alterations for which no approved therapy exists. Therefore, the complexity of data deriving from comprehensive genomic tests and the continuous expansion of molecularly based therapies represent a challenge that requires a common effort to translate molecular profiles into a clinical opportunity for patients (Table 1). In order to correctly integrate all this information into the clinical pathway, it has been proposed to organize MTB. These multidisciplinary groups collate different expertise to facilitate the interpretation and integration of the molecular data of a patient within a path of genetic guided cancer therapy and to promote the access of patients to innovative therapies within clinical trials or with regulatory instruments [37]. 

CGP is also being evaluated in different trials of immunotherapy for cancer treatment. ICIs are widely used in a variety of cancer types, although robust biomarkers predictive of response are still lacking. ICIs have been approved in different lines of therapy in advanced NSCLC. The use of pembrolizumab requires the analysis of the expression of programmed death-ligand (PD-L1) by means of immunohistochemistry (IHC), and the identified cut-offs are an expression level above 50% for first line therapy and above 1% in second lines. For the use of nivolumab and atezolizumab in second line, PD-L1 testing is not mandatory, and no other approved biomarker exists to stratify patients for immunotherapy [38]. The use of PD-L1 as a biomarker poses some challenges in its whole use in clinics. Some points were discussed in the meeting in order to clarify the clinical applicability of the molecular tests in general and the use of PD-L1 as biomarker in particular. 

The analysis of PD-L1 expression provides the exact expression value of the ligand and the range of expression, based on the cut-off value, which leads to the stratification of patients into three groups: patients with negative PD-L1 expression, those with PD-L1 expression level between 1 and 49%, and those with PD-L1 expression ≥50%. The combination of nivolumab and low-dose ipilimumab demonstrated a superior benefit in terms of OS compared to chemotherapy in first-line NSCLC patients whose tumors express PD-L1 ≥1% [39]. In the group of patients with PD-L1 expression ≥50%, the exact expression value might be useful to choose between a regimen with chemotherapy, immunotherapy and bevacizumab, versus pembrolizumab, or even versus chemotherapy alone in specific situations. In fact, patients with tumors with a PD-L1 expression of >90% seem to show significantly improved outcomes with immunotherapy as a single agent, and in these cases the chemotherapy and immunotherapy combination could be avoided (where available; for instance, in Italy it is not reimbursed in the setting of PD-L1 >50%) [40,41].

On the other hand, in the second line scenario where the optimal approach should be evaluated between immunotherapy or docetaxel with or without nindetanib, the PD-L1 negativity or the exact expression value in the 1-49% group might be useful for a retrospective evaluation more than in the clinical practice. However, the extension of immunotherapy combined with chemotherapy in first-line setting reduces this issue to limited cases. 

The second important issue is the material used for PD-L1 evaluation. Indeed, since it is common practice to carry out the PD-L1 test on a cytological sample provided as cyto-included, biopsy-takers should be asked to acquire a histological sample, in order to give patients the opportunity to access clinical trials, where cell-blocks are not accepted. 

However, CGP could also provide relevant information for clinical research in lung cancer patients who are candidates for treatment with ICIs. In particular, TMB is being considered as a possible biomarker, as an alternative to or in association with PD-L1 testing, to stratify NSCLC patients for immunotherapy [42]. TMB is independent from PD-L1 expression, and has been associated with response to ICIs in different clinical studies. However, it is a common belief that at present, the use of TMB to predict the efficacy of the nivolumab plus ipilimumab combination in NSCLC is premature, as has also been suggested by recent results. Furthermore, retrospective analyses conducted on metastatic NSCLC patients enrolled in KEYNOTE-021, -189 and -407 trials showed no significant association between TMB and the efficacy of pembrolizumab plus chemotherapy [43,44,45]. Also, the role of TMB as a predictive factor of response to the durvalumab-tremelimumab immunotherapy combination, suggested by the MYSTIC trial, [46] was not confirmed in the prospective NEPTUNE phase III randomized trial [47]. The role of TMB as a biomarker could also be evaluated in a phase III prospective clinical trial for small-cell lung cancer (SCLC), to identify patients eligible for immunotherapy, as suggested from the results of the CheckMate 032 clinical trial [48].

It must be pointed out that different studies have used different methods and cutoff values for measuring TMB, partially explaining the inconsistency of the obtained outcomes of the clinical trials [49]. A need for the standardization of the methods used, of the mutations to be considered and the cutoffs to define patients with high or low TMB is mandatory in case of more investigations.

To summarize, current evidence suggests that TMB is not a biomarker for the combination of immune checkpoint inhibitors and chemotherapy, while its role in patients receiving immune checkpoint inhibitors as single agents or combinations is still under investigation. In this respect, recent data suggest that TMB might be associated with other biomarkers, such as PD-L1 expression and T-cell–inflamed gene-expression profile (GEP), to better stratify patients for immunotherapy and identify immunological behaviors that can guide therapeutic responses [50,51,52].

Finally, CGP can reveal specific genetic alterations such as mutations in STK11, KEAP1, JAK1/2 and other genes that might affect response to ICIs, thus improving the possibility of tailored treatment of NSCLC patients [9].

## 5. Conclusions

In conclusion, the panel of experts strongly agreed that CGP should be offered to all metastatic non-squamous NSCLC patients in order to improve the use of precision-personalized medicine in this setting and offer more therapeutic opportunities (Table 1). The approval of compounds targeting molecular alterations in a cancer-agnostic scenario, such as small molecules targeting NTRK fusions, further highlights the necessity to introduce CGP in a routine diagnostics flow. 

Apart from the information provided by the analysis of these biomarkers, it would be important to produce genomic profiling data in almost all patients at diagnosis. Indeed, the possibility to have a comprehensive molecular characterization would give the clinician more information on tumor behavior and, beyond the clinical context, it would represent an opportunity for the patient to be enrolled in clinical trials, particularly for those genetic alterations that are present at very low frequencies within the population of cancer patients. 

In this respect, an effort should be made to increase the quantity and the quality of biopsy material available for molecular profiling. In fact, liquid biopsy should be taken into consideration only when tissue is not available, or for monitoring the molecular evolution of the disease. Although PD-L1 is the only biomarker available for immunotherapy, CGP can provide relevant information on sensitivity to checkpoint inhibitors that, in the near future, will be essential for appropriate treatment selection in NSCLC.

## Figures and Tables

**Table 1:** Summary of the indications of the AIOT Expert Panel.

Items	Problems Discussed	Indications
**The sample**	Need for reliable data in a cost and time effective manner. Need to optimize the use of samples for histological and molecular analyses. Need for standardization of operational procedures. Need for an increased efficiency of the sample management.	Centralization of the more complex techniques Optimization of the resources Implementation of multidisciplinary activities Improvement of the training activities Activation of the procedures to spread the figure of the pathologist with specific expertise in lung cancer
**The test**	Informed consent and privacy	Updated for CGP analysis; a shared format should be adopted.
	Tissue versus liquid biopsy	Tissue is mandatory at diagnosis and onset and should be considered the golden standard. Liquid biopsy useful for real-time monitoring of the therapies; its use should be carefully evaluated.
	Comprehensive genomic profiling (CGP) technologies	CGP should be considered after appropriate clinical evaluation and assessment of the adequacy of the sample material. CGP should be performed in specialized laboratories.
	Organization of a laboratory that performs CGP tests	Prerequisites: – structural adequacy; – availability of latest-generation CE-IVD certified instruments and of orthogonal technologies for validation; – qualified and trained personnel; – compliance of the standard operating procedures (SOPs) for the management and traceability of samples and data.
	Outsourcing or in-house testing and CGP validation/verification	In-house tests require: – extensive internal validation of non-certified tests; – participation to external quality assessment schemes; – periodic evaluation of test performances. Test outsourcing requires: – verification that the tests ensure specific quality criteria
	Result interpretation	Implementation of molecular tumor boards (MTBs); planning of a dedicated pathway for patients with germline mutations.
	Mutational report	A structured CPG report must include. – pathological evaluation of the sample; – details on the methods used for testing; – appropriate reporting of the obtained results; – biological and pathological interpretation of the findings
**The clinical applicability**	Use of CGP analysis upfront: when and how	CGP should be offered to all metastatic non-squamous NSCLC patients, to give the patients the access to targeted drugs within clinical practice and in clinical trials.

## Appendix A

AIOT Expert Panel:

- Alessandro Morabito, Thoracic Medical Oncology, Istituto Nazionale Tumori, “Fondazione G. Pascale”—IRCCS, Napoli, Italy. alessandromorabito1@virgilio.it.
- Lucio Crinò, Medical Oncology, Istituto Scientifico Romagnolo per lo Studio e la Cura dei Tumori (IRST) IRCCS, via Maroncelli, Meldola (FC), Italy. lucio.crino@irst.emr.it.
- Domenico Galetta, SSD Oncologia Medica Patologia Toracica IRCCS Oncologico Giovanni Paolo II, Viale Orazio Flacco, 65, 70124, Bari, Italy. galetta@teseo.it.
- Antonio Rossi, Division of Medical Oncology, Fondazione IRCCS “Casa Sollievo della Sofferenza”, San Giovanni Rotondo (Foggia), Italy. arossi_it@yahoo.it.
- Lucio Buffoni, Oncology Department, San Luigi Hospital University of Turin, Orbassano, Turin, Italy. buffonil75@gmail.com.
- Filippo de Marinis, Division of Thoracic Oncology, European Institute of Oncology, IRCCS, Milan, Italy. filippo.demarinis@ieo.it.
- Francesco Grossi, Medical Oncology Unit, Fondazione IRCCS Ca’ Granda Ospedale Maggiore Policlinico, Milan, Italy. francesco.grossi@policlinico.mi.it.
- Federico Cappuzzo, Department of Oncology and Hematology, AUSL Romagna, Ravenna, Italy. f.cappuzzo@gmail.com.
- Cesare Gridelli, Division of Medical Oncology, “S. G. Moscati” Hospital, Avellino, Italy. cgridelli@libero.it.
- Andrea Ardizzoni, Medical Oncology Unit, Sant’Orsola-Malpighi Hospital, Bologna, Italy. andrea.ardizzoni@aosp.bo.it.
- Stefano Gasparini, Pulmonary Diseases Unit, Azienda Ospedali Riuniti, Ancona, Italy. stefano.gasparini@ospedaliriuniti.marche.it.
- Alfonso Marchianò, Department of Radiology, Fondazione IRCCS Istituto Nazionale dei Tumori, Milan, Italy. alfonso.marchiano@istitutotumori.mi.it.
- Juliana Guarize, Division of Thoracic Surgery, European Institute of Oncology, Milan, Italy. juliana.guarize@ieo.it.
- Giovanni Galluccio, Endoscopy, San Camillo Forlanini Hospital, Rome, Italy Email: g.galluccio@scamilloforlanini.rm.it.
- Rocco Trisolini, Interventional Pulmonology Unit, Policlinico Sant’Orsola-Malpighi and Ospedale Maggiore, Bologna, Italy, rocco.trisolini@aosp.bo.it.
- Nicola Fusco, Division of Pathology, Fondazione IRCCS Ca’ Granda - Ospedale Maggiore Policlinico, University of Milan, Milan, Italy. nicola.fusco@unimi.it.
- Mauro Papotti, Department of Oncology, University of Turin, at Città della Salute Hospital, Torino, Italy. mauro.papotti@unito.it.
- Antonio Marchetti, Laboratory of Diagnostic Molecular Oncology, Center for Advanced Studies and Technology (CAST), University of Chieti, Chieti, Italy. amarchetti@unich.it.
- Nicola Normanno, Cell Biology and Biotherapy Unit, Istituto Nazionale Tumori, “Fondazione G. Pascale” - IRCCS, Napoli, Italy. nicnorm@yahoo.com.
- Massimo Barberis, Department of Pathology, European Institute of Oncology, Milan, Italy. massimo.barberis@ieo.it.
- Paolo Graziano, UOC di Anatomia Patologica, Fondazione IRCCS Casa Sollievo della Sofferenza, San Giovanni Rotondo, FG, Italy. p.graziano@operapadrepio.it.
- Simonetta Buglioni, Department of Pathology, “Regina Elena” National Cancer Institute, Rome, Italy. simonetta.buglioni@ifo.gov.it.
- Giulio Rossi, Pathology Unit, Azienda USL Romagna, St. Maria delle Croci Hospital, Ravenna, Italy. giurossi68@gmail.com.
- Giancarlo Pruneri, Department of Pathology, IRCCS Istituto Nazionale Tumori, Milan, Italy; University of Milan, School of Medicine, Milan, Italy. giancarlo.pruneri@istitutotumori.mi.it.
- Daniele Calistri, Biosciences Laboratory, Istituto Scientifico Romagnolo per lo Studio e la Cura dei Tumori (IRST) IRCCS, Meldola, Italy. daniele.calistri@irst.emr.it.
